# Diagnostic Potential of Zymogen Granule Glycoprotein 2 Antibodies as Serologic Biomarkers in Chinese Patients With Crohn Disease

**DOI:** 10.1097/MD.0000000000001654

**Published:** 2015-10-23

**Authors:** Shulan Zhang, Ziyan Wu, Jing Luo, Xuefeng Ding, Chaojun Hu, Ping Li, Chuiwen Deng, Fengchun Zhang, Jiaming Qian, Yongzhe Li

**Affiliations:** From the Department of Rheumatology and Clinical Immunology, Peking Union Medical College Hospital, Chinese Academy of Medical Sciences and Peking Union Medical College, Key Laboratory of Rheumatology and Clinical Immunology, Ministry of Education (SZ, ZW, CH, PL, CD, FZ, YL); Department of Gastroenterology, Peking Union Medical College Hospital, Beijing (JQ); Division of Rheumatology, Department of Medicine, The Second Hospital of Shanxi Medical University, Shanxi (JL); and Clinical Laboratory, General Hospital of CNPC, Jilin, China (XD).

## Abstract

The need for reliable biomarkers for distinguishing Crohn disease (CD) from ulcerative colitis (UC) is increasing. This study aimed at evaluating the diagnostic potential of anti-GP2 antibodies as a biomarker in Chinese patients with CD. In addition, a variety of autoantibodies, including anti-saccharomyces cerevisiae antibodies (ASCA), perinuclear anti-neutrophil cytoplasmic antibodies (PANCA), anti-intestinal goblet cell autoantibodies (GAB), and anti-pancreatic autoantibodies (PAB), were evaluated.

A total of 91 subjects were prospectively enrolled in this study, including 35 patients with CD, 35 patients with UC, 13 patients with non-IBD gastrointestinal diseases as disease controls (non-IBD DC), and 8 healthy controls (HC). The diagnosis of IBD was determined based on the Lennard-Jones criteria, and the clinical phenotypes of the IBD patients were determined based on the Montreal Classification.

Anti-GP2 IgG antibodies were significantly elevated in patients with CD, compared with patients with UC (*P* = 0.0038), HC (*P* = 0.0055), and non-IBD DC (*P* = 0.0063). The prevalence of anti-GP2 IgG, anti-GP2 IgA and anti-GP2 IgA, or IgG antibodies in patients with CD was 40.0%, 37.1%, and 54.3%, respectively, which were higher than those in non-IBD DC (anti-GP2 IgG, 15.4%; anti-GP2 IgA, 7.7%; and anti-GP2 IgA or IgG, 23.1%) and those in patients with UC (anti-GP2 IgG, 11.4%; anti-GP2 IgA, 2.9%; and anti-GP2 IgA or IgG, 14.3%). For distinguishing CD from UC, the sensitivity, specificity, positive predictive value (PPV) and positive likelihood ratios (LR+) were 40%, 88.6%, 77.8%, and 3.51 for anti-GP2 IgG, 37.1%, 97.1%, 92.9%, and 13.0 for anti-GP2 IgA, and 54.3%, 85.3%, 79.2%, and 3.69 for anti-GP2 IgA or IgG. For CD diagnosis, the combination of anti-GP2 antibodies with ASCA IgA increased the sensitivity to 68.6% with moderate loss of specificity to 74.3%. Spearman's rank of order revealed a significantly positive correlation of anti-GP2 IgG with ileocolonic location of disease (L3) (*P* = 0.043) and a negative correlation of anti-GP2 IgA with biologic therapy (*P* = 0.012).

Our findings suggest that anti-GP2 antibodies could serve as a biomarker for distinguishing patients with CD from patients with UC, and the combination of anti-GP2 antibodies with ASCA IgA may improve the predictive power.

## INTRODUCTION

Inflammatory bowel disease (IBD) is a group of chronic relapsing intestinal inflammation of unknown etiology and heterogeneous clinical symptoms and course.^1^ A combination of genetic, environmental, and immunological mechanisms has been proposed to cause and/or contribute to IBD.^1–3^ Crohn disease (CD) and ulcerative colitis (UC) are the 2 major clinical phenotypes of IBD.^1,2^ Both CD and UC present a series of symptoms and signs, including intestinal and extra-intestinal involvements.^1–3^ However, CD and UC display substantial difference in terms of lesion location in the gastrointestinal (GI) tract. Specifically, CD can affect any part of the GI with the lesion formation in the entire bowel wall, whereas UC only affects large intestine with the lesion formation restricted to the epithelial lining of the gut.^1–3^ The different characteristics between CD and UC result in different clinical managements and therapies, especially when it comes to surgical interventions.^1–3^ In addition, diagnostic dilemma can also come from other disorders affecting the GI, which may present similar symptoms to those seen in IBD patients.^1–3^ Therefore, accurate diagnosis is essential for proper clinical interventions.

A number of serological biomarkers have been identified for distinguishing IBD from non-IBD and for distinguishing CD from UC. Anti-saccharomyces cerevisiae antibodies (ASCA) and perinuclear anti-neutrophil cytoplasmic antibodies (pANCA) have been widely used as routine tests for patients with clinical suspicion of IBD.^4,5^ However, the sensitivity of ASCA in CD patients is far from satisfactory.^4^ Recent data showed that the sensitivity of ASCA was 46.3% in Chinese patients with CD.^4^ Interestingly, 2 studies suggested the prevalence of ASCA was much lower in Chinese patients with CD in terms of either ASCA IgA^6^ or ASCA IgG,^7^ challenging the role of ASCA in the diagnosis of different subtypes of IBD. Taken together, these studies indicate a strong need for additional biomarkers to improve the diagnostic sensitivity and accuracy.

Pancreatic autoantibodies (PAB) have been recognized as CD-specific biomarkers.^8–10^ It has been reported that PAB can be detected in approximately 30% of patients with CD but less than 5% of patients with UC or non-IBD and health controls.^9–10^ However, detection of PAB exclusively depends on indirect immunofluorescence (IIF) on pancreatic tissues. Thus, the clinical utility of PAB has been hampered due to its unidentified antigenic targets. Excitingly, zymogen granule glycoprotein 2 (GP2) was recently described as the major autoantigen of CD-specific PAB. GP2 is a highly glycosylated protein, accounting for around 40% of the zymogen granule membrane proteins of pancreatic acinar cells.^11^ Importantly, overexpression of GP-2 has been identified in the intestinal tissue in patients with CD, but not in patients with other immune-mediated enteropathies, such as UC, suggesting a direct involvement of GP2 in the inflammatory process in CD.^11^ In addition, GP-2 was found on the surface of microfold (M) cells of the intestinal Peyer's patches (PP),^12^ which have been considered the original location of CD inflammation.^13^

**FIGURE 1:**
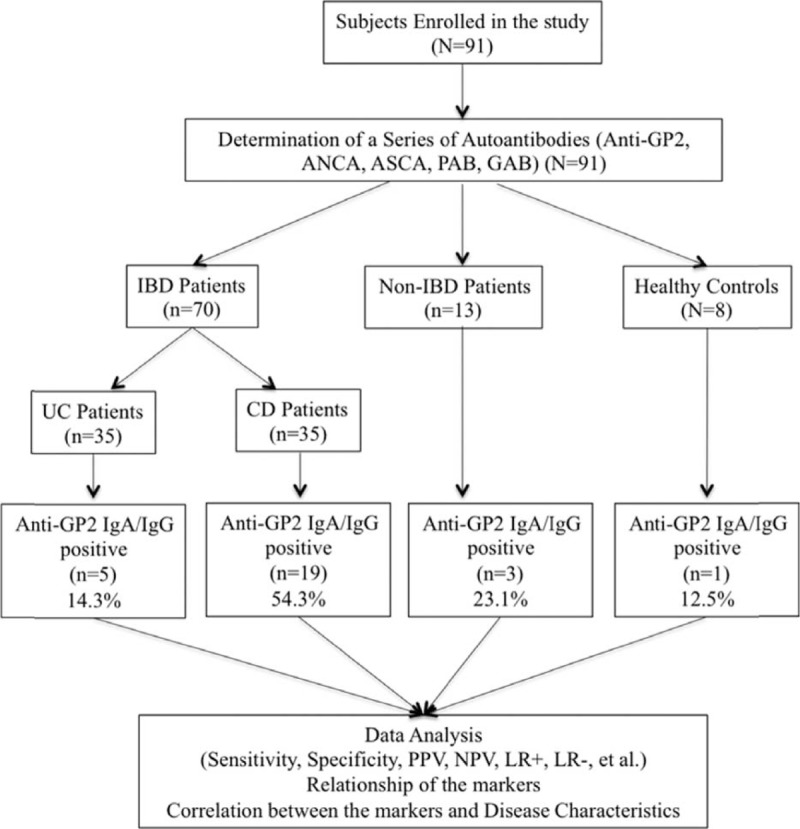
Evaluation of multiple autoantibodies, including anti-GP2, ANCA, ASCA, PAB, GAB antibodies in the diagnosis of Chinese patients with CD.

Recent studies indicate that anti-GP2 IgA and/or IgG were present in 21–45% of patients with CD and in 2–19% of patients with UC.^11,14–18^ Most importantly, 8–24% of ASCA-negative CD patients were positive for anti-GP2 IgA and/or IgG,^16–17^ suggesting that anti-GP2 IgA and/or IgG could be a promising biomarker for distinguishing CD from UC, and the combination of ASCA with anti-GP2 IgA and/or IgG could synergistically strengthen the overall performance. Of note, the prevalence and diagnostic performances can be affected by a variety of factors, and ethnic/geographic background is an important one among these factors. Thus, it is of paramount importance to assess the diagnostic potential of anti-GP2 IgA and/or IgG as a biomarker in Chinese patients with CD. In this study, we determined the sensitivity, specificity, and positive and negative predictive values (PPV and NPV) of a variety of autoantibodies, including anti-GP2 antibodies, ASCA, anti-PAB, pANCA, and anti-intestinal goblet cell autoantibodies (GAB), and investigated their individual diagnostic values as well as combinational diagnostic values in distinguishing CD from UC (Figure 1).

## METHODS

### Subjects and Specimen Collections

A total of 91 subjects were prospectively enrolled in this study, including 35 patients with CD, 35 patients with UC, 13 patients with non-IBD gastrointestinal diseases as disease controls (non-IBD DC), and 8 healthy controls (HC). The non-IBD DC included patients with intestinal Behcet disease (n = 5), intestinal tuberculosis (n = 6), ischemic colitis (n = 2), and infectious colitis (n = 2). HC included subjects without any signs of infection or inflammation or other significant illnesses. All patients were diagnosed and managed at the Department of Gastroenterology, Peking Union Medical College Hospital (PUMCH). The diagnosis of IBD was determined based on the Lennard-Jones criteria.^19^ Specifically, subjects were diagnosed with CD or UC based on a combination of standard criteria that included clinical symptoms, physical examination, colonoscopy, imaging (bariums studies and CT enterography), and histopathology. Enteric infections, intestinal tuberculosis, ischemia, nonsteroidal anti-inflammatory drug-induced ulceration, and radiation colitis were excluded. Clinical phenotypes of the IBD patients were determined based on the Montreal Classification.^20^ Specifically, CD is described by A, L, and B classifications. A represents age at diagnosis (A1, below 17 yr; A2, between 17 and 40 yr; A3, above 40 yr), L represents the location of disease (L1, ileal; L2, colonic; L3, ileocolonic; L4, upper disease), and B represents disease behavior (B1, nonstricturing, nonpenetrating; B2, stricturing; B3, penetrating; P, perianal disease modifier). UC is described by E classifications (E1, proctitis, lesions limited to the rectum; E2, left-sided colitis, lesions below the splenic flexure; E3, pancolitis, lesions exceeded the splenic flexure). The activity of UC was defined by the Simple Clinical Colitis Activity Index (SCCAI) as mild (3–5 scores), moderate (6–11 scores), and severe (above 12 scores). The demographics and clinical characteristics of the CD and UC patients are shown in Table 1. Study protocols were reviewed and approved by the Ethical Committee of PUMCH and informed consents were obtained from all participants. All sera were stored at −20°C until analysis.

**TABLE 1:**
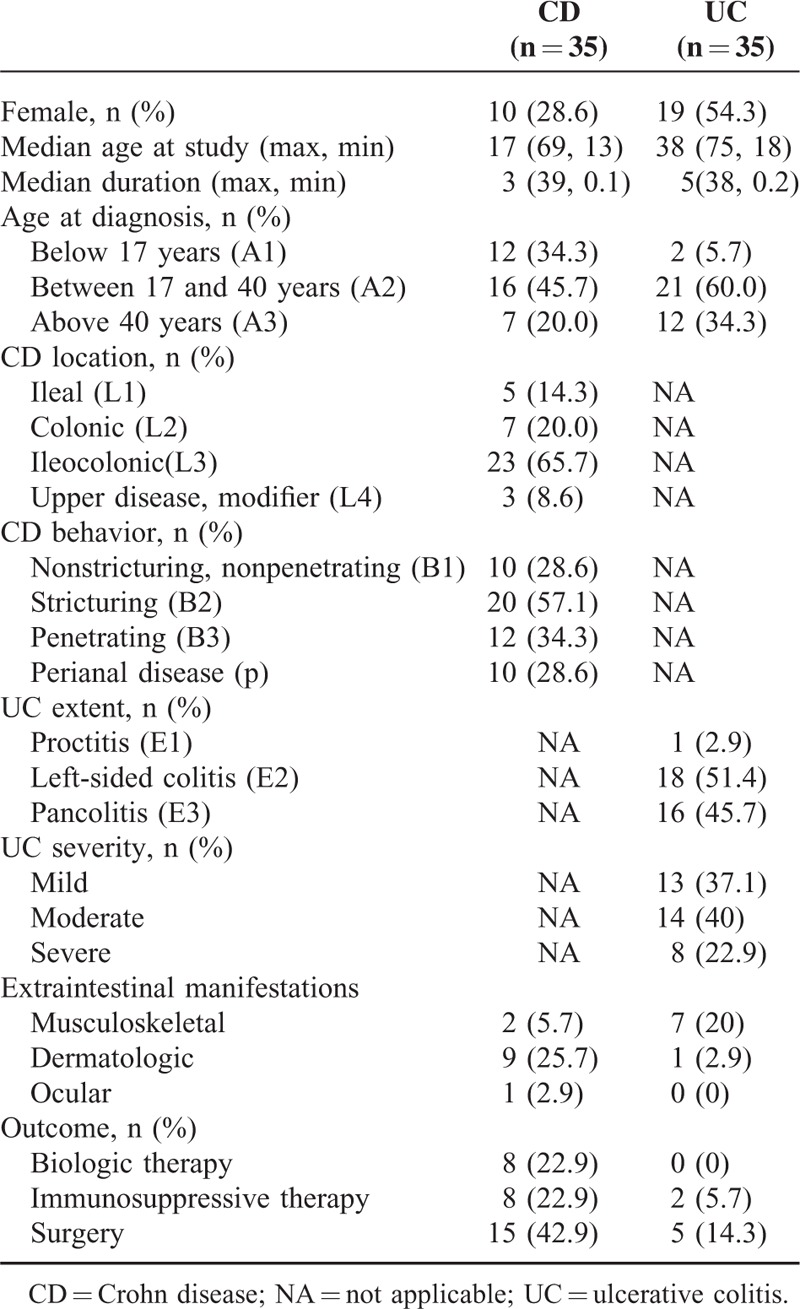
Demographics of Patients With Inflammatory Bowel Disease

### Serum Antibodies Determination

Serum anti-GP2 autoantibodies (IgG and IgA) were determined by ELISA (Generic Assays, Dahlewitz/Berlin, Germany), according to the manufacturer's instructions. The cutoff value for positivity was set to 20 U/mL for both anti-GP2 IgG and anti-GP2 IgA antibodies, as recommended by the manufacturer. Serum anti-saccharomyces cerevisiae antibodies (ASCA) (IgG and IgA) were determined by ELISA (Euroimmune, Luebeck, Germany). Values above 20 U/mL were considered positive according to the manufacturer's instructions. Serum anti-neutrophil cytoplasmic autoantibodies (ANCA) (IgG and IgA), anti-intestinal goblet cell autoantibodies (GAB), and pancreatic autoantibodies (PAB) were tested by indirect immunofluorescent assay (IFA) (Euroimmune, Luebeck, Germany), in accordance with the manufacturer's instructions. IFA testings were performed starting with an initial dilution of 1/10. Serial dilutions of 1/20, 1/40, 1/80, and 1/160 were further performed for all positive samples. Two experienced technologists interpreted the results.

### Statistical Analysis

SPSS 20.0 statistical software package (SPSS Inc, Chicago, IL) and Prism 5.02 (GraphPad Software, San Diego, CA) were utilized for statistical analyses. For comparison of continuous variables, the independent t-test or Mann–Whitney *U* test was performed. For comparison of categorical variables, the *χ*^2^ test or Fisher exact test was employed. The association of anti-GP2, ASCA, or anti-PAB antibodies with disease characteristics was assessed by Spearman's rank of order. For all statistic analyses, *P* values of less than 0.05 were considered statistically significant.

## RESULTS

### Levels of Anti-GP2 IgG Antibodies Were Significantly Elevated in Patients With CD

**FIGURE 2:**
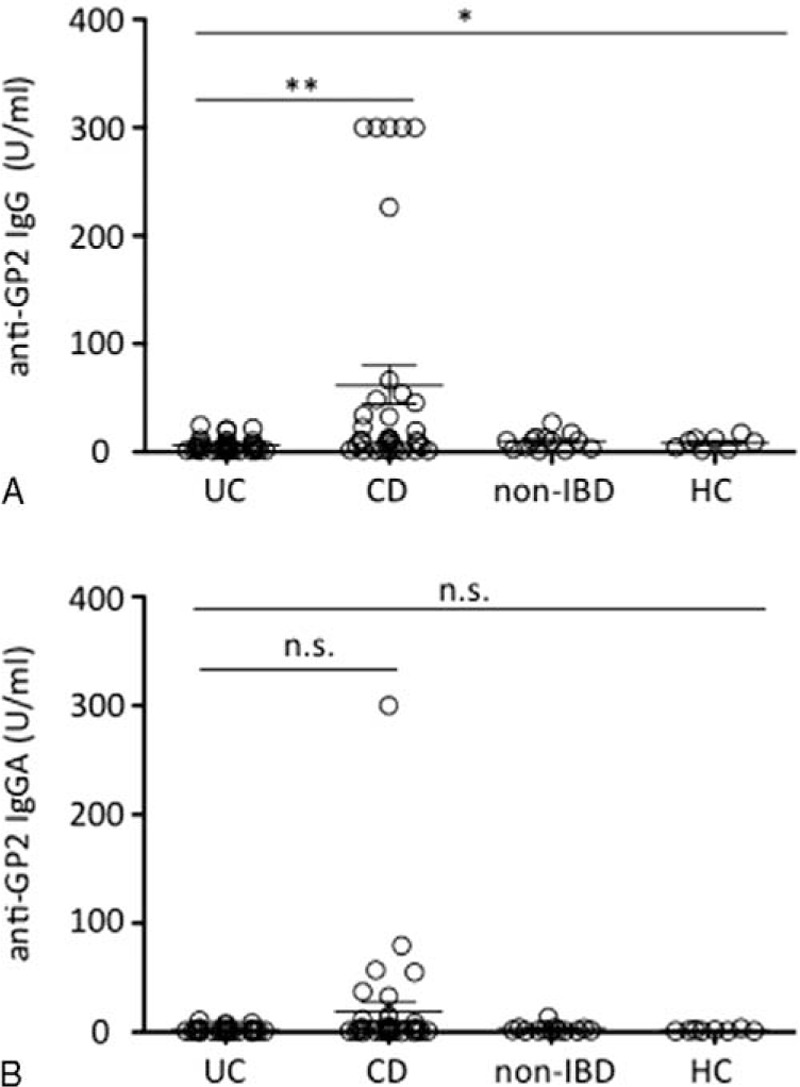
Anti-GP2 IgG (A) and anti-GP2 IgA (B) antibodies levels in patients with UC, patients with CD, non-IBD patients, and healthy controls (HC). n.s. = nonsignificant, ^∗^*P* < 0.05; ^∗∗^*P* < 0.005.

As shown in Figure 2A, anti-GP2 IgG antibodies were significantly elevated in patients with CD, compared with patients with UC (*P* = 0.0038), HC (*P* = 0.0055), and non-IBD DC (*P* = 0.0063). However, no significant difference was observed in anti-GP2 IgA antibodies in patients with CD, compared with patients with UC (*P* = 0.0704), HC (*P* = 0.0834), and non-IBD DC (*P* = 0.0616) (Figure 2B), although a trend of increased levels of anti-GP2 IgA was observed in patients with CD (Figure 2B).

### Prevalence of Multiple Autoantibodies in Patients With CD and UC

The prevalence of a series of IBD-relevant autoantibodies was evaluated among all the subjects, and the results are summarized in Table 2. Overall, the prevalence of anti-GP2 IgG, anti-GP2 IgA, and anti-GP2 IgA or IgG antibodies in patients with CD was 40.0%, 37.1%, and 54.3%, respectively, which were higher than those in non-IBD DC (anti-GP2 IgG, 15.4%; anti-GP2 IgA, 7.7%; and anti-GP2 IgA or IgG, 23.1%) and those in patients with UC (anti-GP2 IgG, 11.4%; anti-GP2 IgA, 2.9%; and anti-GP2 IgA or IgG, 14.3%) (Table 2). Anti-PAB antibodies were detected in patients with CD (IgG, 8.6%; IgA, 5.7%, and IgA or IgG 8.6%) and patients with UC (IgG, 8.6%; IgA, 2.9%, and IgA or IgG, 8.6%), but not in non-IBD DC and HC. ASCA IgA antibodies were present in 25.7% of CD patients, 5.7% of UC patients, 23.1% of non-IBD DC, and 12.5% of HC, respectively. The prevalence of ASCA IgA or IgG antibodies was 25.7%, 11.4%, 30.8%, and 12.5% in CD patients, UC patients, non-IBD DC, and HC, respectively. For the ANCA IgA or IgG antibodies, the prevalence was similar among CD patients, UC patients, and non-IBD DC, ranging from 34.3% to 40.0%. GAB IgA or IgG antibodies were detected in 37.1% of CD patients, 37.1% of UC patients, 15.4% of non-IBD DC, but not in HC. To further assess the potential role of these autoantibodies in distinguishing patients with CD from patients with UC, *P* values were calculated between CD patients and UC patients. A significantly higher prevalence of anti-GP2 IgA or IgG (19/35, 54.3%) was detected in patients with CD, compared with patients with UC (5/35, 14.3%) (*P* = 0.0009). Importantly, the prevalence of both anti-GP2 IgG and anti-GP2 IgA was significantly higher in patients with CD than that in patients with UC (anti-GP2 IgG, *P* = 0.013; anti-GP2 IgA, *P* = 0.0006). In addition, the prevalence of ASCA IgA was significantly higher in patients with CD (9/35, 25.7%), compared with patients with UC (2/35, 5.7%) (*P* = 0.045). No significant difference was found in other autoantibodies, either in IgG subtype or in IgA subtype (Table 2).

**TABLE 2:**
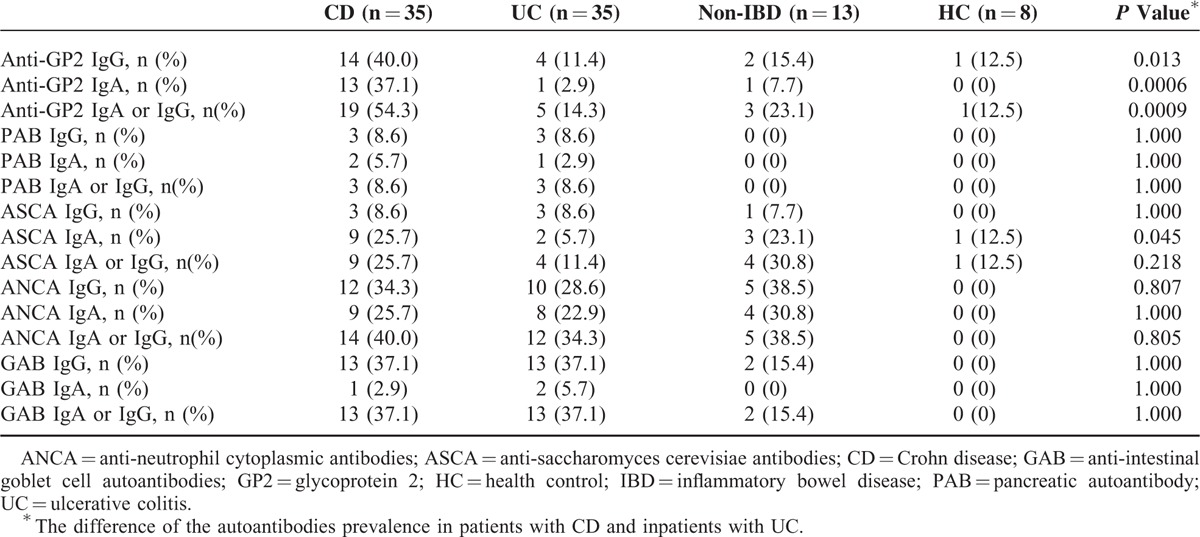
Prevalence of Autoantibodies in Patients With Inflammatory Bowel Disease and Controls

### Predictive Power of Serologic Markers for Distinguishing Patients With CD From Patients With UC

Assay performance characteristics for the detection of anti-GP2 antibodies (IgA and/or IgG) were compared to corresponding ASCA and PAB values, and the results are summarized in Table 3. For distinguishing CD from UC, anti-GP2 IgA or IgG demonstrated the highest sensitivity (54.3%), followed by anti-GP2 IgG (40.0%), anti-GP2 IgA (37.1%), and ASCA IgA or IgG (25.7%) and ASCA IgA (25.7%). The sensitivities of PAB IgA, IgG, IgA or IgG, and ASCA IgG were less than 10% (Table 3). The specificities of all of these markers were similar, ranging from 85.3% to 97.1%. Anti-GP2 IgA showed the highest positive predictive value (PPV) (92.9%) and positive likelihood ratios (LR+) (13.0), followed by ASCA IgA (PPV: 81.8%, LR+: 4.51), and anti-GP2 IgA or IgG (PPV: 72.9%, LR+: 3.69) (Table 3). For distinguishing UC from CD, GAB IgG and GAB IgA or IgG showed the highest sensitivity (37.1%), followed by ANCA IgA or IgG (34.3%), ANCA IgA (22.9%), and ANCA IgG (22.2%) (Table 3).

**TABLE 3:**
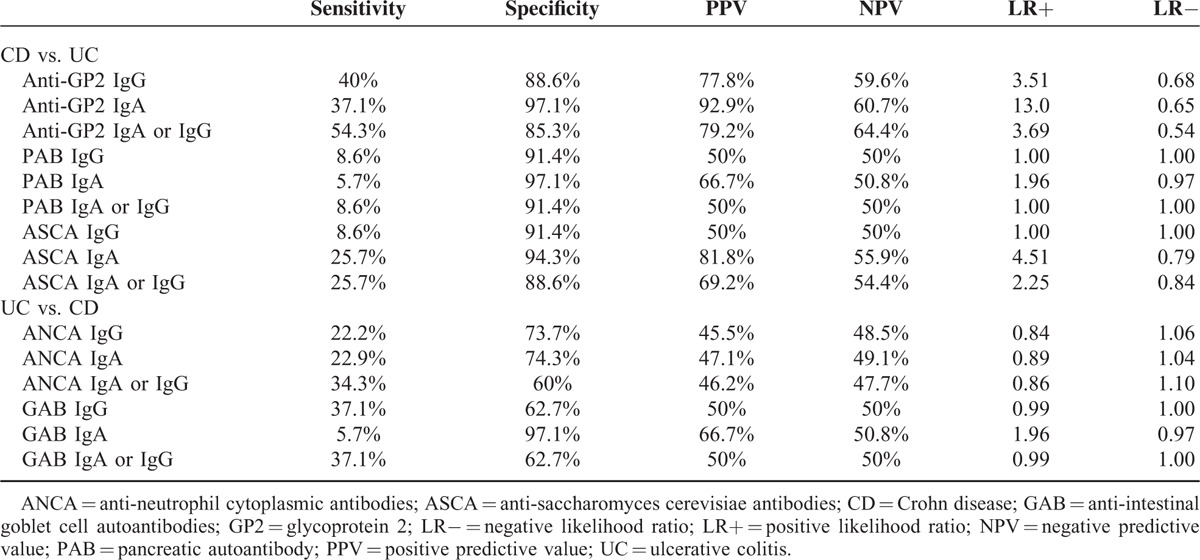
Predictive Power of Serologic Markers for Differentiation Among Patients With Crohn Disease and Ulcerative Colitis

As both anti-GP2 antibodies and ASCA IgA demonstrated a good performance in distinguishing CD from UC, we evaluated the predictive power of combination of anti-GP2 antibodies and ASCA IgA in distinguishing CD from UC. The double positive of anti-GP2 IgA and ASCA IgA, or the triple positive of anti-GP2 IgA, anti-GP2 IgG, and ASCA IgA strikingly raised the specificity and PPV to 100%, but decreased the sensitivity to 8.3% (Table 4). In contrast, either anti-GP2 IgA positive, or anti-GP2 IgG positive, or ASCA IgA positive increased the sensitivity from 54.3% (the sensitivity of anti-GP2 IgA or IgG) to 68.6%, with moderate loss of specificity from 85.3% (the specificity of anti-GP2 IgA or IgG) to 74.3% (Table 4).

**TABLE 4:**
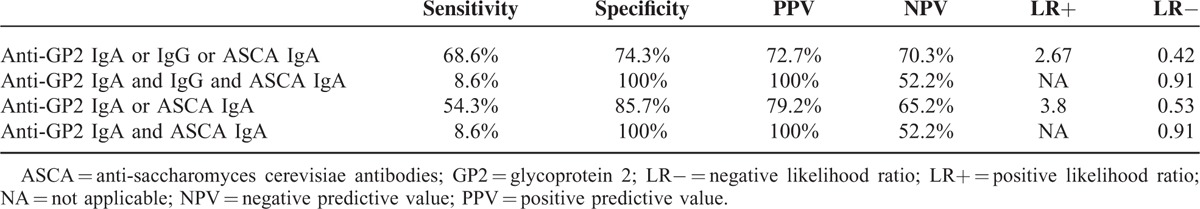
Combined Analysis of Anti-GP2 and ASCA for Differentiation Among Patients With Crohn Disease and Ulcerative Colitis

### Relationships Between Serological Markers (Anti-GP2, ASCA, and Anti-PAB Antibodies) in the CD Cohort and UC Cohort

**FIGURE 3:**
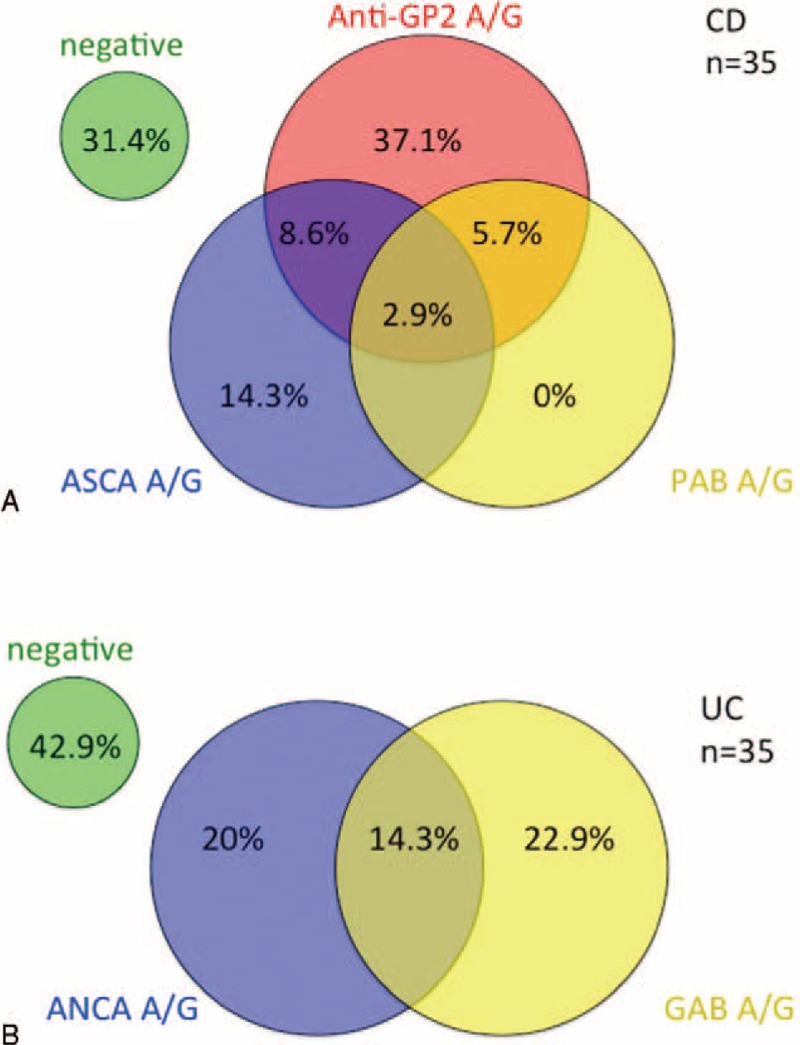
Venn diagram describing the relationships between serological markers (Anti-GP-2, ASCA, and PAB) in the CD cohort (n = 35) by presence vs. absence (A), Venn diagram describing the relationships between serological markers (ANCA and GAB) in the UC cohort (n = 35) by presence vs. absence (B). The percentage of positive patients for each marker, or any combination of 2 markers, is shown.

As more than one of the described autoantibodies (anti-GP2, ASCA, anti-PAB) was found in several patients, we illustrate the distribution of these antibodies in CD patients by Venn diagram (Figure 3A). Of note, 31.4% of patients with CD were negative for all of the 3 antibodies, and the remaining 68.6% of patients with CD reacted to at least 1 marker. Only 2.9% of patients with CD were reactive to all of the markers. Importantly, 42.8% of ASCA negative CD patients were positive for anti-GP2 IgA and/or IgG antibodies, whereas only 14.3% of anti-GP2 negative CD patients were positive for ASCA IgA and/or IgG antibodies. The distribution of autoantibodies (ANCA and anti-GAB antibodies) is illustrated by Venn diagram in Figure 3B. Overall, 42.9% of patients were negative for ANCA and anti-GAB antibodies. Approximately 57% of patients with UC were positive for at least 1 marker, and 14.3% of the patients with UC were reactive to both of the markers (Figure 3B).

### Association of Anti-GP2, ASCA, or Anti-PAB Antibodies With Disease Characteristics of Patients With CD

The association of anti-GP2, ASCA, or anti-PAB antibodies with disease characteristics was evaluated in patients with CD. Spearman's rank of order revealed a significantly positive correlation of anti-GP2 IgG with ileocolonic location of disease (L3) (*P* = 0.043) and a negative correlation of anti-GP2 IgA with biologic therapy (*P* = 0.012) (Table 5). ASCA IgG (*P* = 0.011) and ASCA IgA and IgG (*P* = 0.011) were positively correlated with patients with an age less than 17 years at diagnosis. In addition, PAB IgG (*P* = 0.035) and PAB IgA or IgG (*P* = 0.035) were positively correlated with colonic location of disease (L2), and PAB IgA (*P* = 0.003), PAB IgG (*P* = 0.035), PAB IgA or IgG (*P* = 0.035), and PAB IgA and IgG (*P* = 0.003) were positively correlated with nonstricturing, nonpenetrating disease (B1).

**TABLE 5:**
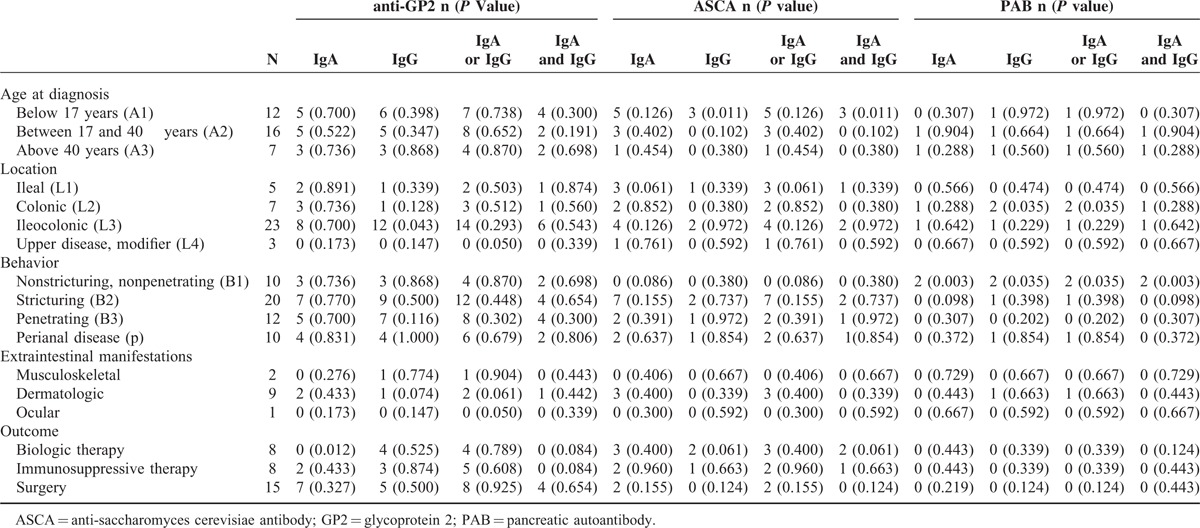
Association of Anti-GP2, ASCA, or PAB Autoantibodies With Disease Characteristics of Patients With Crohn Disease

## DISCUSSION

In this study, we evaluated the prevalence and diagnostic potential of anti-GP2 antibodies in Chinese patients with IBD. The major findings include the following: first, the levels of anti-GP2 IgG antibodies were significantly elevated in patients with CD; second, the prevalence of anti-GP2 (either IgA or IgG subtype) antibodies was significantly higher in patients with CD than that in patients with UC; third, the double positive of anti-GP2 IgA and ASCA IgA, or the triple positive of anti-GP2 IgA, anti-GP2 IgG and ASCA IgA had a strong indication of CD with a specificity and PPV of 100%; fourth, anti-GP2 IgG was positively correlated with ileocolonic location of disease (L3) and anti-GP2 IgA was negatively correlated with biologic therapy. Our findings suggest that anti-GP2 antibodies could serve as a biomarker for distinguishing patients with CD from patients with UC.

Previous studies from several groups in Europe showed that anti-GP2 antibodies were present in 25–30% of patients with CD and in 5–12% of patients with UC.^14,21,22^ In this study, we found that the prevalence of anti-GP2 antibodies was 54.3% in Chinese patients with CD, which is higher than that reported in previous studies.^14,21,22^ However, the prevalence of anti-GP2 antibodies in UC patients in our study is similar to that reported in other studies.^14,21,22^ As different genetic, immunologic, and environmental factors contribute to the pathogenesis of CD, the higher prevalence of anti-GP2 antibodies in Chinese patients with CD could be due to the combination of these factors. More importantly, the increased sensitivity of anti-GP2 antibodies did not sacrifice the specificity, PPV and LR+ values. Therefore, our data suggest that anti-GP2 antibodies could be a promising biomarker for distinguishing CD from UC.

ASCA has been recognized as the most widely used biomarker for CD.^23^ Interestingly, in our study, 42.8% of ASCA-negative CD patients were identified positive for anti-GP2 antibodies (IgA and/or IgG), whereas only 14.3% of anti-GP2-negative CD patients were positive for ASCA (IgA and/or IgG), indicating that anti-GP2 antibodies might be more sensitive in identifying patients with CD. More importantly, the combination of ASCA and anti-GP2 antibodies could synergistically strengthen the power of distinguishing CD from UC. In addition, we noticed that 31.4% of patients with CD were negative for all of the 3 antibodies (ASCA, anti-PAB, and anti-GP2 antibodies), indicating other biomarkers, such as anti-CUZD1 or anti-MZGP2 antibodies,^24,25^ might be helpful in CD diagnosis. Further studies on evaluation of these biomarkers are needed.

GP2 are the specific receptors in the exocrine pancreas as well as on M cells of intestinal Peyer's patches (PP).^12,26^ Interestingly, M-cell-associated GP2 is suggested to be involved in the interaction between the immune system and intestinal microbiota.^27^ Indeed, it has been reported that GP2 can function as an endogenous immunomodulator by modulating both innate and adaptive immune responses.^26–28^ Anti-GP2 antibodies are the autoantibodies targeting GP2, and it has been proposed that anti-GP2 antibodies are generated during ileal inflammation, and the inflamed ileal environment contributes to the release of GP2 by M cells and the continual exposure of GP2 to the immune system.^22^ Consistent with this assumption, Pavlidis et al reported that CD patients with ileal (L1) or extensive disease (L3) presented higher prevalence of anti-GP2 IgG.^16^ We also found anti-GP2 IgG was positively correlated with ileocolonic location of disease (L3). Interestingly, no significant difference on the correlation of anti-GP2 IgG with disease location was observed in Czech Republic CD patients.^29^ Bogdanos et al revealed the association between anti-GP2 IgG with structuring behavior (B2) and perianal disease in CD patients.^17^ However, no significant difference of anti-GP2 IgG with structuring behavior (B2) and perianal disease was observed in our study with Chinese CD patients.

Several limitations in this study need to be pointed out. First, the sample size of our study was small, which may introduce analysis bias. Further studies with large cohorts are needed. Second, the subjects in our study were from a single institution, and these subjects were homogenous Han Chinese ethnic group. A multicenter study with various ethnic groups is needed to evaluate the generalizability of our results.

In summary, our data suggest that anti-GP2 antibodies could serve as a biomarker for distinguishing patients with CD from patients with UC, and the combination of anti-GP2 antibodies with ASCA IgA may improve the predictive power.
