# Diagnostic Potential of Zymogen Granule Glycoprotein 2 Antibodies as Serologic Biomarkers in Chinese Patients With Crohn Disease: Erratum

**DOI:** 10.1097/01.md.0000479947.63919.bc

**Published:** 2016-01-22

In the article “Diagnostic Potential of Zymogen Granule Glycoprotein 2 Antibodies as Serologic Biomarkers in Chinese Patients With Crohn Disease”,^1^ which appeared in Volume 94, Issue 42 of *Medicine*, Dr. Yongzhe Li was not originally credited as a corresponding author in the article. The article has since been corrected online.

## References

[1] Zhang S Wu Z Luo J Diagnostic potential of zymogen granule glycoprotein 2 antibodies as serologic biomarkers in Chinese patients with Crohn disease. *Medicine* 2015 94:e1654. 26496271 10.1097/MD.0000000000001654 PMC4620836

